# Integrating Functional Imaging and Molecular Profiling for Optimal Treatment Selection in Neuroendocrine Neoplasms (NEN)

**DOI:** 10.1007/s11912-023-01381-w

**Published:** 2023-02-24

**Authors:** Grace Kong, Emma Boehm, Owen Prall, William K. Murray, Richard W. Tothill, Michael Michael

**Affiliations:** 1 Department of Molecular Imaging and Therapeutic Nuclear Medicine, Peter MacCallum Cancer Centre, 305 Grattan Street, Melbourne, VIC 3000 Australia; 2 The Sir Peter MacCallum Department of Oncology, The University of Melbourne, Melbourne, VIC Australia; 3 Centre for Cancer Research and Department of Clinical Pathology, University of Melbourne, Melbourne, VIC Australia; 4 Department of Pathology, Peter MacCallum Cancer Centre, Melbourne, VIC Australia; 5 Department of Medical Oncology, Peter MacCallum Cancer Centre, Melbourne, VIC Australia

**Keywords:** Neuroendocrine neoplasms (NEN), Neuroendocrine tumors (NET), PET/CT, Functional imaging, Molecular profile, Genomics

## Abstract

**Purpose of Review:**

Gastroenteropancreatic NEN (GEP-NEN) are group of malignancies with significant clinical, anatomical and molecular heterogeneity. High-grade GEP-NEN in particular present unique management challenges.

**Recent Findings:**

In the current era, multidisciplinary management with access to a combination of functional imaging and targeted molecular profiling can provide important disease characterisation, guide individualised management and improve patient outcome. Multiple treatment options are now available, and combination and novel therapies are being explored in clinical trials.

**Summary:**

Precision medicine is highly relevant for a heterogenous disease like NEN. The integration of dual-tracer functional PET/CT imaging, molecular histopathology and genomic data has the potential to be used to gain a more comprehensive understanding of an individual patient’s disease biology for precision diagnosis, prognostication and optimal treatment allocation.

## Introduction

Neuroendocrine neoplasms (NENs) are an exemplar of the rational application of diagnostic modalities for characterisation and treatment selection in the age of precision medicine. NENs are a diverse group of malignancies arising from epithelial cells with neuronal differentiation and secretory capacity as part of the diffuse endocrine system. The term “neuroendocrine neoplasm” encompasses the well-differentiated neuroendocrine tumours (NETs) and the poorly differentiated neuroendocrine carcinomas (NECs), which have differing pathogenesis, behaviour and prognosis [1]. NEN is histopathologically divided into grade 1 NET (G1, Ki-67 < 3%), grade 2 NET (G2, Ki-67 3–20%), grade 3 NET (G3, Ki-67 > 20%), poorly differentiated NEC (small cell/large cell subtypes, Ki-67 usually > 55%) and mixed neuroendocrine-non-neuroendocrine neoplasm (MiNEN) [2••]. NEN most commonly arises from the gastrointestinal tract and pancreas (approximately 65%), collectively called gastroenteropancreatic NEN (GEP-NEN). The overall incidence of NEN is rising, currently, approximately 7.0–9.7 cases per 100,000 depending on geography [3–5]. NEN should thus be elevated from its historical “rare cancer” status to the “uncommon” cancer category, hence requiring increasing awareness.

Due to non-specific hormone secretory syndromes or symptomatology, NEN is often identified late: 60–85% of patients have incurable metastatic disease at diagnosis [2••, 6]. It is at this advanced stage that patients are typically referred for multidisciplinary assessment. Initial workup of NEN typically involves conventional radiology and histopathology assessments but these alone are inadequate to provide full characterisation for this complex heterogenous disease. This review will focus on the importance of precision evaluation and the need to improve and develop diagnostic paradigms to guide personalised therapeutic treatment of GEP-NEN. Access to molecular imaging and molecular testing can resolve diagnostic uncertainty, aid prognostication and guide therapeutic selection particularly for patients with higher-grade disease where disease heterogeneity is common. We will discuss the important role of molecular imaging with positron emission tomography (PET) using somatostatin receptor (SSTR) tracers, integrated with metabolic imaging using 2-[18F]fluoro-2-deoxy-d-glucose [^18^F]FDG (FDG) to non-invasively assess disease biology and heterogeneity. In addition, the development and integration of molecular testing with pathway-focussed histopathological analysis and both germline and tumour somatic mutational analysis can provide further important diagnostic insights, as well as treatment stratification for selected patients with GEP-NEN [2••].

## Molecular Imaging: a Non-invasive Way to Understand Whole Body Disease Biology and Guide Treatment Selection

Radiology using computed tomography (CT) and magnetic resonance (MRI) remain the cornerstone of NEN imaging and are widely available for detecting and monitoring sites of disease. However, recognised limitations of CT include the inability to identify small malignant primary NEN lesions, lymph nodes or bone metastases which are prevalent for metastatic NEN [7••, 8, 9••]. The sensitivity and specificity for NEN detection or restaging may be reduced if serial scanning is performed using non-uniform protocols [10]. It is now well established that molecular PET/CT imaging using SSTR and FDG radiotracers play essential incremental roles in the staging, restaging and theranostics selection for patients with NEN, by characterising specific disease biology.

### SSTR PET/CT Imaging

SSTR (particularly subtype 2) is commonly overexpressed on well-differentiated NEN and represents a useful molecular imaging and therapeutic target [11, 12•]. The initial approved modality [^111^In]In-DTPA-octreotide single-photon emission computed tomography (SPECT)/CT has become superseded by PET/CT imaging due to its superior imaging resolution, diagnostic performance and quantitation [13–15]. Even sub-centimetre lesions with high SSTR expression can be visualised with a high target-to-background ratio. Currently available FDA-approved SSTR-targeting PET radiotracers include [^68^ Ga]Ga-DOTATATE, [^68^ Ga]Ga-DOTATOC and [^64^Cu]Cu-DOTATATE. Existing guidelines from the European Neuroendocrine Tumor Society (ENETS) [7••], European Association of Nuclear Medicine (EANM) [8] and Society of Nuclear Medicine & multi-society workgroup for Molecular Imaging Appropriate Use Criteria [9••] support SSTR imaging for NEN diagnosis, initial staging after histologic diagnosis, pre-surgical assessment, treatment monitoring especially for NEN lesions seen predominantly on SSTR PET, and detection of recurrent disease and importantly for selection of patients for SSTR-targeted Peptide Receptor Radionuclide Therapy (PRRT).

### FDG PET/CT Imaging

FDG is the most used oncological PET imaging agent. Uptake of this radiolabeled glucose analogue correlates with tissue metabolism and proliferation, where uptake is typically high in rapidly growing tumours or tumours with metabolic reprogramming favouring glycolysis. FDG is not a NEN-specific tracer, but FDG positivity is closely correlated with higher NEN tumour grade (typically G2 or G3 NET and NEC), poor differentiation and worse prognosis [16•, 17•, 18, 19]. Studies have established an inverse relationship between proliferation rate and SSTR positivity [20, 21]. A higher proliferation rate is expected for higher-grade disease in approximately 75% of G3 NET and around 90% of NEC cases [22, 23].

### Dual-Tracer Imaging (SSTR and FDG Tracers)

This combined imaging approach can provide powerful complementary information to characterize NEN biology. It is well recognized that significant heterogeneity can exist within an individual patient, such that well-differentiated lesions (SSTR-expressing) can co-exist with higher-grade components (often FDG-avid) [24, 25]. SSTR imaging positivity is a marker of well-differentiated NEN. FDG positivity is a marker of disease metabolic activity and NEN aggressiveness. The use of dual-tracer imaging can assess the heterogeneity of disease biology within a patient, impacting on prognostication and management.

#### As a Prognostic Biomarker

Typically, patients with FDG positive/SSTR negative disease have a poor prognosis and shorter overall survival compared to patients with FDG positive/SSTR positive, or solely SSTR positive disease respectively (latter with best prognosis) [26•, 27]. Earlier institution of more aggressive treatments and frequent monitoring is warranted for patients with highly FDG-avid disease.

#### To Guide Biopsy Site

Tumour grading based on ease of access or location alone may not be representative of the true highest-grade disease given the potential disease heterogeneity. Dual-tracer imaging phenotype can guide the site for targeted biopsy. Typically, the lesion with the most intense FDG uptake is likely to represent a disease of the highest proliferative activity and grade [28].

#### To Guide Patient Management and Therapeutic Approach

Molecular imaging phenotype guides selection for PRRT and stratifies other systemic therapies. High SSTR expression at all disease sites is the main prerequisite for PRRT. PRRT can be effective even if lesions show FDG uptake provided that all these lesions also have high SSTR expression to allow therapeutic targeting [29, 30]. Spatially discordant (FDG positive/SSTR negative) disease cannot be targeted with PRRT alone, and in this case, other systemic or combination options should be considered [26•, 31]. Given the poorer prognosis, patients with highly FDG-avid disease (concordant or discordant) should be followed up more frequently following therapy.

The dual-tracer molecular imaging approach is therefore highly recommended for patients with (1) higher-grade disease including G2 and 3 NEN; (2) patients with presumed G1 disease but with non-SSTR-avid suspicious lesions on radiological imaging; (3) at the time of more rapid progression than expected for the grade (i.e. initial pathological sampling error or transformation to higher grade); (4) to assess heterogeneity and guide biopsy site; and (5) for theranostic selection and to guide therapeutic options [12•].

Whilst SSTR imaging is now widely considered the standard of care for NEN, the combined use with FDG PET/CT is yet to be universally applied due to geographical differences in resources and regulatory limitations. Its benefits warrant further prospective validation to enable integration in NEN management.

## Histopathology: Defining Morphology and Protein Expression for Diagnosis and Prognostication

Histopathological evaluation of tumour morphology, proliferative index and immunohistochemical (IHC) biomarker expression is the foundation of NEN diagnosis and grading [1, 2••, 6]. As discussed, the use of molecular imaging phenotype will guide the biopsy site to ensure sampling representative of the highest-grade lesion. Guidelines specify a minimum requirement for structured reporting of morphology, immunostaining for expression of standard neuroendocrine differentiation markers (chromogranin A, synaptophysin and CD56 or INSM1), as well as proliferation markers (Ki-67/MIB1) (1). GEP-NENs are almost always pan-cytokeratin-positive, but CK7/CK20-negative. The use of morphology and proliferative index to stratify GEP-NENs into NETs (G1-3) or NECs has prognostic and therapeutic implications; however, the optimal parameters remain controversial, and predictors of treatment response are lacking [32]. Importantly, the assessment of the Ki-67 index may be limited by sample error due to inadequate sample size or scoring methodology and should be performed by pathologists with experience in NENs to ensure accuracy and reproducibility. Patients with GEP-NET G3 have better overall survival (OS) than patients with NEC at 43.6 vs 5.3 months [33]. Patients with NEC have been reported to have a better response to platinum-based chemotherapy than NET-G3, although overall survival remains lower [33]. It is important to recognise however that classification based on morphology alone may be challenging and molecular analysis is an essential adjunct.

IHC markers of neuroendocrine cell-of-origin and differentiation are essential to resolve the common diagnostic uncertainty around defining G3 NET versus NEC. Additional IHC markers of NET differentiation include somatostatin receptor type 2 (SSTR2), which can also be used to infer somatostatin analogue (SSA) sensitivity and utility of SSTR functional imaging and is reduced in poorly differentiated cancers [34, 35]. Nuclear staining for the neuroendocrine transcription factor Insulinoma-associated protein-1 (INSM1) has very high sensitivity and specificity (99 and 96% respectively) for GEP-NET, and 100% positive and negative predictive value for differentiating pancreatic NET from other pancreatic differentials including ductal adenocarcinoma, solid pseudopapillary neoplasm and acinar cell carcinoma [36–38]. Loss of immunostaining for alpha-thalassemia/mental retardation X-linked (ATRX) and Death Domain Associated Protein (DAXX, pancreatic NET) correlates with loss of function mutations and is associated with well-differentiated disease and may have prognostic value [34, 39••]. Retained expression of ATRX and DAXX, but the loss of expression of retinoblastoma 1 (RB1) and SMAD4, and altered p53 expression are typical of GEP-NECs [2••, 32, 34, 39••, 40, 41]. Glucose Transporter-1 (GLUT1) positivity is a marker of aggressive behaviour and poor prognosis in GEP-NET [42–44], and a potential surrogate for FDG PET/CT positivity. SSTR2, INSM1, ATRX, DAXX, RB1 and p53 IHC assessments are now more frequently available in anatomical pathology departments and should be incorporated as part of standard care for complex cases unable to be resolved by routine histological examination.

## Genomics of GEP-NEN: a Nuanced Understanding of Individual Disease Biology Has the Potential to Inform Patient-Specific Treatment Strategies

Whilst the integration of molecular imaging and histopathology/IHC techniques have improved NEN characterisation and patient care, a precision medicine approach is needed to manage such complex heterogenous disease and improve individualised outcome. NETs and NECs have distinct genomic profiles and gene drivers (some can be inferred via IHC as in the previous section) such that the role of genomic analysis in GEP-NEN should extend beyond the consideration of germline testing for risk management alone. Rather, genomics can aid in diagnosis, prognosis, treatment selection and trial design.

### Germline Testing

Germline testing is currently only recommended for GEP-NET patients with features of clinical endocrine tumour syndromes [45–48]. It has long been known that approximately 10% of GEP-NEN is associated with germline mutations driving the classical syndromes of multiple endocrine neoplasia type 1 (*MEN1*, encoding the histone modifying Menin 1 protein), as well as neurofibromatosis 1 (*NF1*), von Hippel Lindau (*VHL*) and tuberous sclerosis (*TSC1/TSC2*). To challenge this paradigm, the seminal International Cancer Genome Consortium study involving whole genome sequencing of 98 apparently sporadic pancreatic NETs revealed previously unknown germline alterations in up to 17% of patients including homologous recombination DNA repair genes (*BRCA2* and *CHEK2*) as well as the base-excision DNA repair gene *MUTYH* [49••, 50]. For patients with small intestinal GEP-NET (SI-NET), long been considered a sporadic disease notorious for a paucity of recurrent driver genes (with the exception of somatic *CDKN1B* in a minor fraction), germline mutations in *IMPK*, *OGG1* and DNA repair-associated genes including *CHEK2*, *RAD51C*, *ATM* and *MUTYH* have recently also been identified [50–52]. The pathogenicity and clinical significance of these defects in SI-NET are at present unclear [53, 54••].

Recognising the cohort of patients with GEP-NEN who harbour DNA repair defects and have SSTR-expressing disease on molecular imaging could inform the rational allocation to combination PRRT and drugs that inhibit alternative/rescue DNA repair pathways, such as Poly-ADP Ribose (PARP) inhibitors to maximise radiosensitivity. Such a therapeutic strategy is under active investigation in the PARLuNET trial (NCT05053854), and NCT04086485. Patients with tumours driven by DNA repair defects might also plausibly benefit from a combination of radionuclide therapy and DNA-damaging agents used in the treatment of advanced NET including the antimetabolite capecitabine and the alkylating agent temozolomide [31, 55].

### Somatic Profiling

Genomic profiling of NEN reveals recurrent features and has a clear diagnostic application. NETs typically have few driver mutations [56••, 57••]. Sporadic NETs frequently harbour somatic mutations in *MEN1* but also *VHL* and *TSC2* [58, 59]*.* Loss of function mutations in chromatin-modifying genes *ATRX*/*DAXX* corresponds to alternative lengthening of telomeres (ALT), chromosomal instability and recurrent genome-wide patterns of chromosomal loss [49••, 60, 61, 62••, 63••, 64••, 65]. Mutations in histone modifiers (e.g. *SETD2*, *KMT2C*) and chromatin remodelling genes (e.g. SWI/SNF subunits *ARID1A*, *SMARCA4*) and the PI3K/AKT/mTOR pathway (e.g. *PIK3CA*, *PTEN*, *DEPDC5*) are recurrent in NETs [49••, 60, 66]. *YY1* mutations are enriched in insulinomas [62••, 67]. Some novel gene fusions including EWSR1-BEND2 and NET1-AKR1C3/4 have been reported in GEP-NETs [49••, 63••, 64••). The vast majority of NECs (small and large cell type) harbour mutations in *TP53* plus either *RB1* or *CCNE1* and *MYC* amplifications [63••]. *TP53* mutations are also common in G3NET [63••]. NEC can have tissue of origin mutation patterns, including mutations in *KRAS* (pancreatic NEC), *APC* and *BRAF* (colorectal NEC) [63••], while NOTCH1/2/3 inactivating mutations are enriched in non-pancreatic GI and lung NECs [63••, 68].

Somatic testing can potentially lead to targeted treatment or trial allocation in NEN, and comprehensive genomic profiling is endorsed at clinical discretion in NEN NCCN guidelines [45]. The NCI-MATCH study found that 10% of patients with unspecified subtypes of “neuroendocrine cancer” who underwent tumour panel gene testing were allocated to trials [69]. Studies of somatic mutational testing in cohorts of patients with NEN observed that, depending on the NEN subtype, more than 20% of tumours tested harbour at least one potentially actionable mutation for on-label or off-label therapies as per clinical genomic databases [64••, 70]. Commonly implicated targetable pathways include DNA repair (e.g. *BRCA2*, *ATM*, *RAD51C*); activation of PI3K/Akt1/mTOR signalling and inhibition of the negative PI3K/mTOR pathway regulator PTEN; and amplification of growth factor receptor signalling including *EGFR*, *ERBB2* and *FGFR* [54••, 63••, 64••, 70, 71]. A small proportion of NEN harbour actionable gene fusions including *NTRK* fusions (multiple NEN subtypes) and *ALK* fusions (lung NEN) with case reports of treatment response to entrectanib and alectanib, respectively [72–74]. *MGMT* inactivation via methylation has been demonstrated to occur broadly in NEN; however, the most appropriate *MGMT* promotor methylation assay thresholds for NEN and their use to predict disease response to temozolomide have not yet been clearly established [75–77]. Somatic testing for high tumour mutational burden (TMB; TMB-high > 10 mutations/Mb) can identify patients in whom immune checkpoint inhibitor (ICI) therapy may be effective, though this has been found in only approximately 5–6% of NEN [45, 78•]. TMB-high NEN have been found to harbour defects in DNA repair (MSI, MUTYH-deficiency) or to have smoking-associated (lung NEN) or treatment-associated (alkylating agent) mutational signatures [66].

### Liquid Biopsy

The detection and analysis of circulating tumour DNA (ctDNA) from blood sampling are a non-invasive method to overcome procedural risks and the issues of undersampling of disease heterogeneity inherent in tissue biopsy. Given the limitations in sensitivity and specificity of current markers such as chromogranin A for diagnosis/prognostication in NEN, novel non-invasive biomarkers are sorely needed. Feasibility has been demonstrated by Zakka et al. who undertook ctDNA analysis using Guardant360® assay (73 gene panel) of 320 patients with NEN, finding molecular alterations in 87.5% of patients [79••]. Other novel ctDNA biomarkers under investigation in NEN include copy number change and methylation pattern [80, 81••]. Another approach, the NETest™, is a 51-gene panel detecting circulating tumour RNA, the levels of which are extrapolated to reflect “-omic” biological pathway perturbations reported as a “disease activity score” between 0 and 100% [82]. The NETest™ is not in widespread use due to limited independent validation and assay complexity.

## Treatment Selection for GEP NEN: Current Approach and Future Perspectives for Precision Therapy

The selection of therapy for NENs is currently primarily based on histology (grade), primary site, structural/functional imaging, IHC and clinical behaviour. As described, molecular characterisation (e.g. TMB status) may have a role in future treatment decision-making.

### Grade 1 and 2 NETs—More Indolent Disease

#### First-line Therapy

Somatostatin analogues (SSAs, either depot octreotide or Lanreotide) have demonstrated antisecretory and antiproliferative effects in terms of disease progression, but without significant overall survival benefit [83–85]. The most favourable effect was observed in patients with low hepatic tumour load [83] and in Ki-67 < 10% [85].

#### Second Line and Beyond

PRRT, molecular targeted agents (MTAs: everolimus and sunitinib) and chemotherapy. These are utilised in patients not suitable for SSA, if there is rapid disease progression, or poor prognostic features (high burden, high grade or FDG-avid disease).

For PRRT, patient selection is based upon functional imaging demonstrating high tumour SSTR expression without discordant FDG-avid disease (where performed). Its approval was based on phase III NETTER-1 trial, which demonstrated a 20-month PFS rate benefit in midgut NET in favour of ^177^Lu-DotaTate PRRT (65.2%) versus high dose Octreotide LAR alone (10.8%) [86]. The lack of OS advantage can be explained by high cross over into the PRRT group [87••]. Several series have demonstrated the benefit of PRRT in other primary sites, especially pancreatic NETs [88]. A meta-analysis compared ^177^Lu DOTATATE PRRT with everolimus and observed that the ORR and PFS were greater for PRRT: 47% vs 12% and 25.7 vs 14.7 months, respectively (*P* < 0.001) [89••]. A randomised phase II trial in patients with pancreatic NEN also confirmed the superiority of PRRT versus sunitinib [90••]. The completed COMPETE phase III study has compared ^177^Lu DOTANOC PRRT to everolimus (NCT03049189). Current trials are evaluating PRRT combined with PARP inhibitors (NCT05053854) and capecitabine (NCT02736448). Retreatment of patients with PRRT is feasible, with a recent meta-analysis demonstrating a median PFS of 12.5 months and OS of 26.8 months, with a similar safety profile as initial therapy [91••].

Regarding MTAs, everolimus has demonstrated increased PFS relative to placebo in pancreatic NETs (HR = 035, *P* < 0.001) [92], non-functional pulmonary NETs and GEP NETs (HR = 0.48, *P* < 0.00001) [93]. Sunitinib has a PFS advantage relative to placebo for pancreatic NETs: HR = 0.42, *P* < 0.001 [94].

In terms of chemotherapy, modern phase III trials are lacking. Patients selected are those with progression post-SSA, PRRT or MTAs, if unsuitable for PRRT, or those with large volume or rapidly progressive disease. The integration of dual-tracer molecular imaging plays an important role in identifying patients with these poor prognostic features. Chemotherapy is more active in patients with pancreatic NETs, with ORR from 31 to 70% and OS exceeding 40 months [95•]. Regimens include capecitabine plus temozolomide (CapTem), temzolomide, FOLFOX, capecitabine-oxaliplatin (CapOx) and streptozotocin-5FU. The activity of CapTem was confirmed by the randomised phase II E211 trial [96••].

The optimal therapy sequencing of the available options, however, has not been validated. The SEQTOR study (GETNE 1206) randomised patients with progressive pancreatic NET to everolimus followed by streptozotocin-5FU upon progression (arm A), or the reverse sequence (arm B). On initial analysis, both sequential strategies showed similar efficacy and PFS [97••].

### Grade 3 NETs and NECs

The treatment approach for patients with G3 NETs and NECs differs substantially given their histopathology, imaging characteristics and genomics (see Table 1). Given the more aggressive nature of the disease, early institution of therapy is important to optimise patient outcome: the integration of molecular imaging and molecular profiling could play an important role for these patients.

**Table 1:** Summary of typical clinically relevant GEP-NEN imaging and molecular profiles stratified by grade

	NET grade 1	NET grade 2	NET grade 3	NEC
Disease behaviour				
	Indolent	Intermediate	Aggressive	Very aggressive
PET/CT molecular imaging phenotype(19, 21)				
SSTR-targeted	+ + +	+ + +	+ +	-/ +
FDG	-	-/ +	+ +	+ + +
Histopathology(1, 34, 35, 37, 39, 42)				
Morphology	Well differentiated			Poorly differentiated (small cell/large cell)
Ki-67	< 3%	3–20%	> 20%	> 20% (typically > 55%)
IHC staining				
SYN	Positive (diffuse)	Positive (diffuse)	Positive (diffuse)	Variable^a^
CgA	Variable	Variable	Variable	Variable
INSM1	Positive staining in GEP- NEN vs other: Sensitivity 99%/Specificity: 96%^b^			
GLUT1	+	+ +	+ + / + + +	+ + +
SSTR2	+ + +	+ + +	+ +	±
Other IHC	Some have loss of ATRX or DAXX staining. Retained staining for RB1 and wild-type p53			Retained staining for ATRX and DAXX. Loss of RB1 and/or p53 staining SMAD4 negative SC-NEC: TTF1 positive
Genomic features(49–51, 63, 64, 70, 71)^c^				
Germline mutations	*MEN1*, *NF1*, *VHL*, *TSC1/2*, *CDKN1B*, *MUTYH*, *BRCA2*, *CHEK2*, *ATM*, *RAD51C*, *IMPK* (SI-NET), *OGG1* (SI-NET)			Typically sporadic^d^
Somatic driver pathways/mutations	*MEN1*, *VHL*, *TSC2*,* YY1* Telomere maintenance and ALT (*ATRX*/*DAXX*) Epigenetic modifiers (*SETD2*, *KTM2A*, *ARID1A* others) DNA repair deficiency (*BRCA2*, *ATM*, *RAD51C*, others) PI3K/Akt1/mTOR signalling activation (e.g. *PTEN*, *PIK3CA*) Growth factor signalling (*EGFR*, *ERBB2*, *FGFR*, others) *NTRK*-fusion, *ALK*-fusion (lung) *EWSR1-BEND2*, *NET1-AKR1C3/4*			*TP53*, *RB1* alterations *CCNE1/MYC* amplification *KRAS* (pNEC), *APC*, *BRAF* (CR-NEC) *NOTCH1/2/3* MSI
TMB average^e^ (64, 70)	1.09–4.6			5.1–5.45

*CgA*, chromogranin A; *IHC*, immunohistochemistry, *FDG*, 2-[18F]fluoro-2-deoxy-d-glucose; *SSTR2*, somatostatin receptor 2; *SYN*, synaptophysin; *TMB*, tumour mutational burden (somatic mutations per megabase); *SC-NEC*, small cell NEC; *pNEC*, pancreatic NEC; *CR-NEC*, colorectal NEC

− negative + weak positive +  + moderate positive +  +  + strong positive

^a^SYN may be focal or negative in some NEC

^b^Further evaluation required in G3NET/GEPNEC

^c^Selected list of clinically relevant features

^d^*PALB2* germline mutation reported in pancreatic NEC (68)

^e^Approximately 5% of NEN are TMB-high (> 10 mut/Mb) (66, 67, 76)

#### First-line Therapy

The clinical behaviour of NECs is similar to extensive-stage small cell lung cancer (SCLC) [22]. Treatment is platinum-based (cisplatin/carboplatin) plus etoposide (EP), with median survival ranging from 9.5 to 19 months [98••, 99, 100, 101•, 102, 103] ad a short median PFS from 4 to 6 months with an ORR of 30–50% [98••, 100, 104]. Irinotecan plus cisplatin, based on Japanese randomised trials, showed similar or superior response rates relative to EP [105, 106••]. Differentiation status in G3 disease [102, 107] and a Ki-67 ≤ 60% predict less benefit from platin-based chemotherapy [100]. In G3 NET, the ORR to platin-based regimens is < 5%, with PFS < 3 months, but prolonged OS [18, 100, 108, 109].

Hence, patients with G3 NETs benefit from similar therapies used in G2 NETs [18, 22]. Several heterogeneous retrospective series have indicated activity for CapTem in G3 NETs: ORR varies from 30 to 51%, median PFS of 9 to 15.3 months and OS from 19 to 29.3 months [110–114]. The optimal threshold for higher ORR is a Ki-67 from 10 to 40% [115]. Data for other therapies in G3 NET is limited. The pivotal SSA phase III trials had not included G3NET [83, 116] and so their use should be limited to patients with confirmed SSTR expression, no FDG discordance (this should be closely monitored), or for management of secretory syndromes [117]. The data on MTAs in G3 NET is sparse. Everolimus has been evaluated in patients with G3NETs (Ki-67 20–55%) in the first/second-line setting (*N* = 15), with a median PFS of 6 months, and OS of 28 months [118]. A completed German study (EVINEC) has evaluated everolimus as a second-line treatment for G3 NET and G3 NEC (NCT02113800). Sunitinib was evaluated in 31 patients with pancreatic grade 3 NET/NECs: with partial response in 4 and stable disease seen in 14 patients [119]. A completed Nordic phase II study has evaluated temozolomide and everolimus as first-line treatment in metastatic G3NET (Ki-67 21–55%) (NCT02248012).

#### Second Line and Beyond

Patients with NECs/G3 NETs may benefit from subsequent chemotherapy [100]. Options for G3 NETs include chemotherapy (subject to prior exposure), MTAs (as above) and PRRT. In the case of NEC, patients that have progressed in ≥ 3 months post platinum-based treatment may still be platinum-sensitive (100). Other regimens include FOLFIRI, FOLFOX and CapTem. In terms of Irinotecan-5FU-based regimens, the ORR ranges from 17 to 40%, PFS 4–5.8 months and OS 5–11 months [120–122]. For Oxaliplatin-5FU, PR ranges from 23 to 29%, PFS 4.5 months and OS 9.9 months [123–125]. CapeTem has also demonstrated activity in this setting [113]. However, patients with Ki-67 > 55% have worse outcomes [126]. The SEcond-line therapy in NEuroendocrine CArcinomas (SENECA) phase II study is evaluating FOLFIRI or CAPTEM post failure of first-line chemotherapy in patients with lung and NEC [127].

PRRT is an option, as G3NETs have the greater propensity for SSTR expression relative to NECs: its utility here has been reported by several small studies [31, 128–130, 131••]. In the largest series reported (*N* = 69 G3NET/NEC), the median PFS was 9.6 months, and the median OS was 19.9 months; for patients with Ki-67 ≤ 55% (*n* = 53), the median PFS was 11 months and OS 22 months, for those with Ki-67 > 55% (*n* = 11), 4 months and 7 months, respectively [31]. An analysis of 4 studies where PRRT was used in the second/third line setting: overall PFS was 19 months in G3NET, 11 months for NEC (Ki-67 ≤ 55%) and 4 months for NEC (Ki-67 > 55%) [131••]. Thus, PRRT may be considered for patients in G3 NENs with Ki-67 < 55% [31, 131••]. Current trials include the phase III COMPOSE study of ^177^Lu-DOTANOC versus systemic therapy (NCT04919226) and the NETTER-2 phase III trial randomising patients to PRRT versus high dose SSA (NCT03972488). PRRT is being combined with Nivolumab (NCT04525638).

ICI is also promising in progressive high-grade NET and NEC, based on their higher TMB; the latter is greater in NECs and with microsatellite instability noted in 14% of NECs [132]. A meta-analysis of 10 heterogenous, single-arm studies of ICI in NEN (*N* = 464) found a pooled ORR of 15.5% [133). The response was based on primary site: with thoracic NEN being more likely to respond than GEP-NEN (ORR 24.7% vs 9.5% respectively) and well-differentiated tumours having a lower response rate than NECs (ORR 10.4% vs 22.7% respectively) [133]. Very limited activity has been observed with single-agent immunotherapy [134, 135], relative to combined PD1 and CTL4 blockade. From the CA209-538 study, 29 patients with heavily pre-treated NETs were treated with a combination of ipilimumab and nivolumab. Overall, in the 13 (45%) with high-grade disease, the ORR was 24% and a DCR of 72% [136••]. The SWOG S1609 DART trial reported the results of the high-grade G3 NET/NEC cohort (*N* = 19) with a median Ki-67 value of 80%. The ORR was 26% and the clinical benefit rate (stable disease for ≥ 6 months plus PR and CR) was 32% [137••]. Other trials are yet to be reported, including a phase II trial of PDR001 (PD-L1 inhibitor) (NCT02955069), Nivolumab combined with EP (NCT03980925) and toripalimab in pancreatic NEN (NCT03043664, NCT02939651 and NCT03147404). Even within TMB-high NENs, however, there is a heterogeneous response to ICI highlighting the need for further biomarkers for stratification.

## Perspectives for Precision Therapy Utilizing Multidisciplinary Diagnostic Approaches

**Fig. 1:**
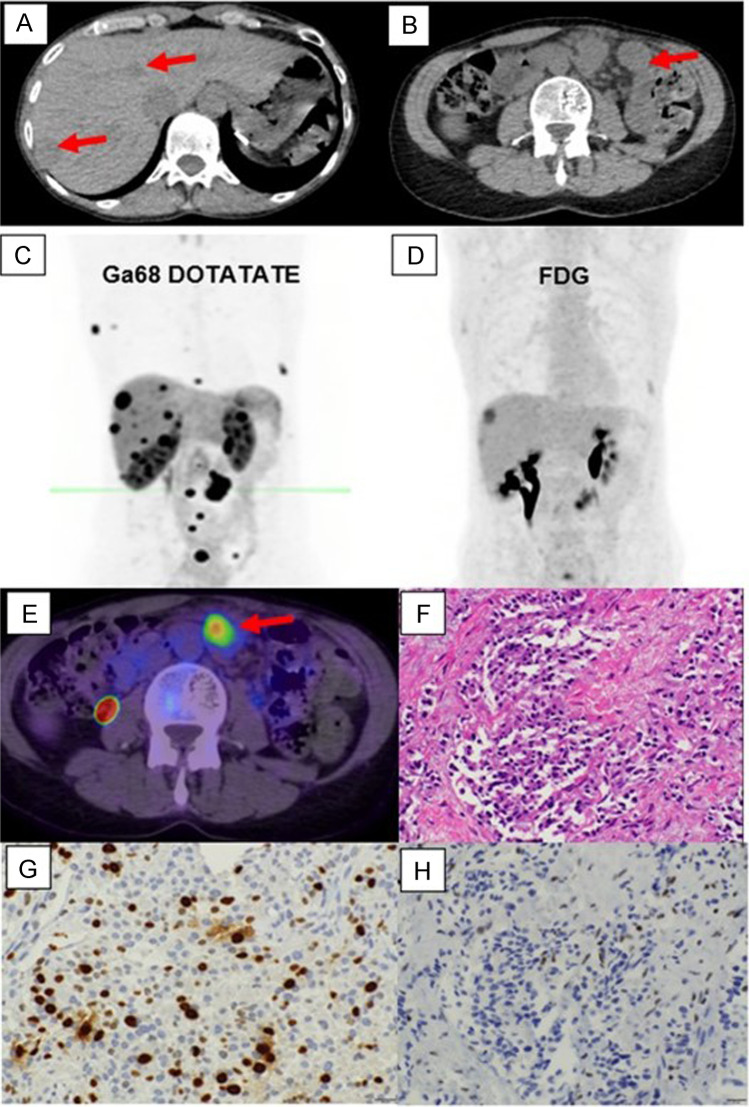
A case example of a 54-year-old female, with a previous history of treated localised breast cancer, and previously resected grade 1 (Ki-67 2%) pancreatic NET. She presented with new, multiple hepatic (**A**) and mesenteric nodal metastases (**B**). A Ga-68 DOTATATE PET/CT (**C**) showed metastatic disease in the liver, nodes and bones with high SSTR expression. FDG PET/CT (**D**) showed some lesions with concordant FDG avidity. The lesion with the highest metabolic activity (mesenteric node, **E**) was targeted for biopsy and diagnosis. Histopathology (**F**) showed monotonous cuboidal cells with granular eosinophilic cytoplasm, ovoid nuclei and fine chromatin. By IHC Ki-67 labelling index was 25% (**G**) and DAXX expression was lost (**H**). Other IHC (not shown) demonstrated expression of SSTR2 and synaptophysin, retained ATRX and Rb, a p53 wild-type pattern, and no staining for chromogranin or multiple breast markers. Overall, the features were supportive of a G3 NET and not breast carcinoma or NEC. Genomic sequencing confirmed DAXX mutation and MEN1 mutation, typical for NET. The patient proceeded to receive PRRT treatment for metastatic G3 NET

NEN is a challenging, heterogenous disease with different clinical, imaging, pathological and genomic complexities to consider in each patient. Multiple treatment options are now available, and combination and novel therapies are being explored in clinical trials. However, clinical treatment selection and sequencing are still mainly based on disease grade, primary site, agent availability and local protocols, without personalisation. Precision medicine is highly relevant for a heterogenous disease like NEN. In the current era, the integration of molecular imaging (SSTR and FDG PET/CT) and molecular profiling (IHC profile and genomic analyses) can provide important disease characterisation, to guide precision management and individualised treatment selection/sequencing (see Fig. 1). This is particularly crucial for patients with advanced high-grade NENs and to resolve G3 NET vs NEC disease biology, as clinical behaviour and treatment options can differ significantly. It is also imperative to focus on incorporating prospective serial translational genomic analysis of tissue and blood, to develop novel liquid biopsy and tumour testing methodologies to understand NEN pathogenesis, discover predictive and prognostic biomarkers to explain the differential response to therapy and subsequently guide future trial design for rational treatment allocation. Using multidisciplinary diagnostic approaches should be the focus of future development to improve individualised therapy and patient outcomes.

## Conclusion

We are in an exciting era for the biological interrogation of neuroendocrine neoplasms to guide precision management by incorporating molecular imaging assessment with clinically relevant molecular pathology pathway and genomic evaluation. Our technological capability for precision diagnosis needs to be developed in parallel with therapy advancements in patients with advanced-stage higher-grade NEN and globally is only a reality for patients who have geographical or financial access to major NEN referral centres [138]. It is therefore imperative not only to place molecular imaging and genomics at the centre of NEN patient management but to also show the symptomatic, survival and health economic benefits of doing so through high-quality research such that these technologies are widely supported by guidelines and imbursed by regulatory bodies.
